# The Arabidopsis *TETRATRICOPEPTIDE THIOREDOXIN-LIKE 1* Gene Is Involved in Anisotropic Root Growth during Osmotic Stress Adaptation

**DOI:** 10.3390/genes12020236

**Published:** 2021-02-07

**Authors:** María Belén Cuadrado-Pedetti, Inés Rauschert, María Martha Sainz, Vítor Amorim-Silva, Miguel Angel Botella, Omar Borsani, Mariana Sotelo-Silveira

**Affiliations:** 1 Laboratorio de Bioquímica, Departamento de Biología Vegetal, Facultad de Agronomía, UdelaR, 12900 Montevideo, Uruguay; belencuadrado1994@gmail.com (M.B.C.-P.); msainz@fagro.edu.uy (M.M.S.); oborsani@fagro.edu.uy (O.B.); 2 Laboratorio de Señalización Celular y Nanobiología, Instituto de Investigaciones Biológicas Clemente Estable (IIBCE), 11600 Montevideo, Uruguay; irauschert@iibce.edu.uy; 3 Departamento de Biología Molecular y Bioquímica, Instituto de Hortifruticultura Subtropical y Mediterránea “La Mayora,” Universidad de Málaga-Consejo Superior de Investigaciones Científicas (IHSM-UMA-CSIC), Universidad de Málaga, Campus Teatinos, 29071 Málaga, Spain; vitoramorimsilva@uma.es (V.A.-S); mabotella@uma.es (M.A.B.)

**Keywords:** TTL1, root growth, Arabidopsis, atomic force microscopy

## Abstract

Mutations in the Arabidopsis *TETRATRICOPEPTIDE THIOREDOXIN-LIKE 1* (*TTL1*) gene cause reduced tolerance to osmotic stress evidenced by an arrest in root growth and root swelling, which makes it an interesting model to explore how root growth is controlled under stress conditions. We found that osmotic stress reduced the growth rate of the primary root by inhibiting the cell elongation in the elongation zone followed by a reduction in the number of cortical cells in the proximal meristem. We then studied the stiffness of epidermal cell walls in the root elongation zone of *ttl1* mutants under osmotic stress using atomic force microscopy. In plants grown in control conditions, the mean apparent elastic modulus was 448% higher for live Col-0 cell walls than for *ttl1* (88.1 ± 2.8 vs. 16.08 ± 6.9 kPa). Seven days of osmotic stress caused an increase in the stiffness in the cell wall of the cells from the elongation zone of 87% and 84% for Col-0 and *ttl1*, respectively. These findings suggest that *TTL1* may play a role controlling cell expansion orientation during root growth, necessary for osmotic stress adaptation.

## 1. Introduction

Drought is a significant constraint for crop production worldwide. A critical trait related with water deficit tolerance is the root adaptation capacity to keep growing and exploring the soil in a new hydric condition [1,2,3,4]. Even though it is critical for plant survival, root growth adaptation during this stressful condition is not well understood [5].

Arabidopsis primary root growth is directed by a small number of stem cells located in the root apical meristem (RAM) that generate all the cell types of the root from cell division followed by regulated cellular expansion and differentiation [6]. In the longitudinal axes, Arabidopsis primary root is organized in 4 developmental zones: the proximal meristem (PM), the transition zone (TZ), the elongation zone (EZ), and the differentiation zone (DZ) [7,8]. The PM extends from the quiescent center (QC) to the first elongated cell; where, isodiametric cells with high mitotic activity began radial expansion defining the root width [8]. In the TZ cells near the PM maintain the mitotic activity while the cells in the distal part develop anisotropic expansion in the longitudinal axis [8]. In the EZ, cells exponentially elongate, with large vacuoles and the nucleus displaced to the cell wall. In this zone, multiple changes occur at the cellular level, such as in microtubules reorientation, cellulose deposition, cell wall softening, and new cell wall synthesis [9]. Fully elongated cells are displaced to the DZ where terminally differentiate and no longer elongate [7,8]. Root growth reaches a stationary regime that is the result of the delicate balance of cell division, elongation, and differentiation, in which the regulation of the direction and extent of cell wall expansion of EZ cells play a major role [10,11,12]. In the stationary phase, the sizes of the PM and EZ of the root remain constant and the root grows by increasing the length of the DZ, a process that is in turn dictated by the mature cell length and the cell proliferation rate in the meristem [7,10].

During the past few years, several studies emphasized the role of brassinosteroids (BR) in directing root growth [13,14,15,16,17]. BR deficient mutants have short roots due to less elongated mature cells. Moreover treatments with high BR concentrations inhibited root growth due to a reduction in the size of meristem produced by an acceleration in cellular elongation [17]. In the EZ, BR promotes cell elongation through BRASSINAZOLE-RESISTANT 1 (BZR1) transcription factor activation, and endogenous levels of BR are required to maintain the balance between the meristematic zone and the elongation zone. It was shown that the BZR1 expression pattern and subcellular localization depend on endogenous BR and auxin levels [12], and that auxin distribution is also influenced by the BR content [18], establishing opposed gradients along the root meristem. Besides the opposed distribution gradients of auxins and BR, evidence derived from transcriptomic analysis of root apices showed that they have opposite effects on cellular elongation and the control of genes that co-regulate this process [12,13,17,18]. Therefore, the spatiotemporal antagonism between auxins and BR is important to maintain cellular domains of quiescence, division and elongation that lead to an equilibrated root growth rate [12,19,20].

Despite these advances in the understanding of the mechanism of root growth, little is known about how the balance between cell division and elongation leading to root growth is maintained during challenging environmental conditions [21]. It has been shown that the primary root responds to saline stress dynamically; immediately after transfer to hyperosmotic media, the root growth rate drops due to a reduction in turgor pressure [15]. Depending on the stress severity, cells in the elongation zone may enter a quiescent stage before their growth rate is recovered [15,22]. The timing of growth recovery was predicted to be regulated by brassinosteroid and gibberellic acid signaling pathways that regulate genes involved in cell wall synthesis [15,22]. At the cellular level, growth is regulated by turgor pressure and the rate of cell wall loosening. Under hyperosmotic stress, root epidermal and cortex cells swell [23] similar to that observed with genetic or chemical disruption of cell wall organization [24,25,26,27,28]. Likewise, mutants impaired in cell wall organization/integrity are hypersensitive to osmotic/ionic stress [22,29,30]. At the organ level, hyperosmotic stress inhibits root cell proliferation and elongation [23].

Mutation in the Arabidopsis *TETRATRICOPEPTIDE THIOREDOXIN-LIKE 1* (*TTL1*) gene cause root swelling and root growth arrest under NaCl and osmotic stress [31,32,33]. *TTL* genes encode a novel Arabidopsis family of proteins specific to land plants that have six tetratricopeptide repeat (TPR) domains located in specific positions of the sequence and a sequence with homology to thioredoxins in C-terminal position [31,32]. TPR domains are well-described protein–protein interaction modules. Recently, Amorim-Silva et al., 2019 showed interaction between TTL3 and constitutively active BRI1, BSU1, and BZR1. Moreover TTL3 has been associated in vivo with most BR signaling components except for BRI1-ASSOCIATED KINASE1 (BAK1). TTL3 showed dual cytoplasmic and membrane localization dependent on endogenous BR content suggesting that TTL proteins may function as positive regulators of BR signaling [33]. In this work we investigated the role of *TTL1* in primary root growth in osmotic stress conditions. We aimed to understand if the swelling phenotype observed in osmotic stress conditions is due to the involvement of *TTL1* in the maintenance of the physical properties of the cell wall during the anisotropic cell expansion process that contributes to the final organ size.

We show that a mutation in *TTL1* caused a deceleration in root growth rate compared to Col-0 with increasing osmotic potential. Moreover, osmotic stress caused both a specific inhibition in the elongation of cells in EZ as well as a reduction in the number of cortical cells in the PM.

Atomic force indentation experiments showed that the cell wall of cells in the EZ of *ttl1* were more elastic than those of Col-0 even in control conditions. Under osmotic stress, both the cell wall of the elongation zone cells of Col-0 and those of *ttl1* became stiffer, but *ttl1* cell walls did not reach the magnitude of stiffness of Col-0 cells walls. These data together with the reduction in cell elongation and proliferation observed in the *ttl1* mutant under osmotic stress support a role for *TTL1* in maintaining cell wall physical properties necessary for sustaining a proper root growth during osmotic stress.

## 2. Materials and Methods

### 2.1. Plant Material and Growth Conditions

We used the original T-DNA insertion lines Salk_063943 (for *TTL1*; AT1G53300) that is in the *Columbia-0* (Col-0) wild-type background. *ttl1*, *pTTL1::GUS* and the *Procuste1 (prc1-1)* mutant, which consist in a knock-out mutation (Q720stop) in the *CESA6* gene (AT5G64740), and was previously described in [34,35]. *35S::BZR1::YFP* seeds [17] were kindly provided by Zhi-Yong Wang.

The seeds were surface sterilized with 70% (*v*/*v*) ethanol for 7 min, followed by 20% sodium hypochlorite for 7 min, and washed three times with sterile water. Seeds were stratified at 4 °C in the dark for 4 d and plated onto petri dishes containing basal Murashige and Skoog (MS) medium [36] + sucrose 1.5%, for in vitro germination Plates were placed in a long-day regime (16 h of light/8 h of dark) with 25 µmol m^2^ s^−1^ light intensity and 23 °C (day/night).

### 2.2. Osmotic Stress Treatment

Seedlings were grown 5 d in basal MS + 1.5% sucrose medium (−0.4 MPa) and then transferred to petri dishes containing basal MS + 1.5% sucrose supplemented with 300 mM mannitol (−0.7 MPa) or 400mM mannitol (−1.2 MPa). Osmotic potential was estimated by cryoscopic osmometer model OSMOMAT 030 (Gonotech, Berlin, Germany). Seedling were grown under stress conditions for 7 days, unless stated otherwise.

### 2.3. Polymerase Chain Reaction (PCR)-Based Genotyping

Identification of the *ttl1* allele was undertaken using the following primers: LBSALK (5′-TGG TTC ACG TAG TGG GCC ATC G-3′); TTL1DPCRF (5′-TGG ACT CAC CACCACCAC TA-3′) and TTL1DPCRR (5′-ACC GAG TCT GCG AAC AAG AT-3′)

### 2.4. Epibrasinolide and Propiconazole Sensitivity Assay

To test root growth inhibition by epibrasinolide (BL) and propiconazole (PCZ) seedlings were germinated in basal medium (MS + 1.5% sucrose) as described previously. After 5 days of growth in vertical plates, the seedlings were transferred to basal medium supplemented with 0.1 µM of BL, 0.2 µM BL or 0.5 µM PCZ; or hyperosmotic medium (MS + 1.5% sucrose + 400mM mannitol) supplemented with 0.1 µM of BL, 0.2 µM BL or 0.5 µM PCZ. After 9 days in each treatment roots were photographed, and root length was measured using Zeizz-ZEN pro Imaging Software (Zeizz, Oberkochen, Germany). Comparisons between genotypes in different treatments were determined by a Student’s *t*-test, in all cases a *p* < 0.05 was considered significant.

### 2.5. GUS Staining Assay

For GUS analysis, Arabidopsis roots were incubated overnight at 37 °C with a 5-bromo-4-chloro-3-indolyl-β-glucuronic acid solution (Gold Biotechnology Inc., St. Louis MO, US) plus 1 mM K_3_Fe(CN)_6_ y 1 mM K_4_Fe(CN)_6_·3H_2_O. For clearings, tissue was treated overnight with Hoyer’s solution [37] plus 20% lactic acid. Light images from GUS-stained tissues and from roots were obtained using Zeizz Axio Imager M2 microscope (Zeizz, Oberkochen, Germany) equipped with differential interference contrast (Nomarski) optics.

### 2.6. Root Meristem Analysis

To measure meristem characteristics, we follow the protocol of [38]. Root proximal meristem size was determined by counting the number of cortex cells in a file extending from the QC to the first elongated cell excluded. EZ extended from the first elongated cell till the first hair in the epidermis layer. Mature cell length was measured in the last cell of the EZ. Comparison between the number of cortical cells in the PM and mature cell lengths of Col-0 and the mutants were determined by a Student’s *t*-test, in all cases a *p* < 0.05 was considered significant. Images used to measure were taken using Zeizz Axio Imager M2 microscope and processed with Zeizz-ZEN pro Imaging Software.

The root length from plants growing in vertical plates was measured over time from 5 to 10 days after germination; 30 roots of each genotype were measured from the hypocotyl to the root tip. We used “least squares” method to determine the curve of best fit for each root length over time. The root growth rate was calculated from the slope of the regression for each root. The root growth rate of Col-0 and the mutants in each growth condition were determined by a Student’s *t*-test, in all cases a *p* < 0.05 was considered significant.

An estimate of growth parameters from cortical cell length profiles were calculated as described by [39].

- Root growth rate: root length at day 8—root length at day 6
- Cell production rate: a /average mature cell length
- Length of the cell cycle: number of cortical cells in PM/b * ln (2)
- The average time interval between each cortical cell leaving the meristem to enter the TZ and EZ: 1/b
- Elongation cell rate in the PM: ½ max cell length in PM/c

### 2.7. Atomic Force Microscopy (AFM) Nanoindentation Experiments

Atomic force microscopy (AFM) indentation analysis were conducted with an atomic force microscope (BioScope Catalyst, Bruker, Billerica, MA, USA).

Seedlings were attached to Petri dishes with a thin layer of silicone glue (PEGAMIL, ANAEROBICOS S.R.L., Buenos Aires, Argentina).

The plated seedlings were placed into the optical inverted microscope coupled to the AFM (Olympus IX81, Miami, FL, USA). Roots from 7 days seedlings studied using an AFM fluid cantilever holder at 25 °C. The absence of PI fluorescence was used to confirm the viability of the roots, and only live roots were measured. All AFM measurements were performed within 1 h after insertion of the AFM head. The silicon nitride probe (DNP-10, *cantilever A*; Bruker, Billerica, MA, USA), with a tip radius of 20 nm, was attached to a triangular 175-μm-long cantilever with a spring constant of 0.35 N/m, following the manufacturer’s instructions. For indentation we did as follows: (1) the cantilever was positioned using a ×10 and ×20 magnification eyepiece on the surface of the fourth elongated epidermal cell of the primary root; taking care to position it in the center of the cell. A low (1 Hz) frequency was set to maximize the number of force curves that were captured, as previously described [40]. At least 150 force curves were captured for each root.

Young’s Modulus (AEM) was obtained from first and second-order polynomial fits of the obtained force curves, according to [41].

All force curves were fitted with a second-order polynomial to obtain the Young’s Modulus. Force curves with a correlation coefficient lower than 0.99 were discarded. A normalized histogram of the remaining data was created and fitted with a Gaussian distribution. Data points outside the 95% confidence interval were discarded and both the histogram and the Gaussian fit were recalculated.

All comparisons between groups were determined by a Student’s *t*-test, in all cases a *p* < 0.05 was considered significant.

Topography images were obtained with MSNL-10/A probe in contact mode.

### 2.8. Epistasis

For *TTL1* and *CESA6*, we evaluated the level of epistasis by comparing the AEM of *ttl1 × prc1-1* double mutant with the product of the AEM *ttl1* and AEM *prc1-1* values of the corresponding single mutants [42] using this formula: ε = AEM *ttl1 × prc1-1* − AEM *ttl1* AEM *prc1-1* [42], in which ε = 0 describes no epistasis, ε > 0 corresponds to buffering epistasis and ε < 0 to aggravating epistasis [42].

### 2.9. Confocal Imaging

Fluorescence signal of *35S::BZR1::YFP* and *ttl1 35S::BZR1::YFP* was observed with confocal microscopy Zeizz LSM 800—AiryScan (Zeizz, Oberkochen, Germany). Exitation was done using a 488 nm laser, and emission was detected between 505 and 550 nm.

## 3. Results

### 3.1. Root Growth Rate Decelerate When Osmotic Potential Increases

**Figure 1:**
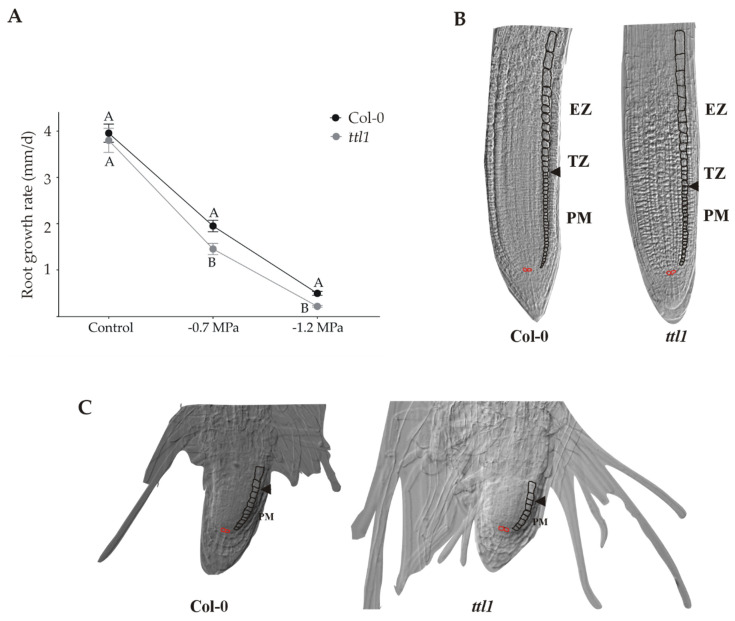
The *ttl1* mutant exhibits greater reduction of root growth rate than Col-0 in response to increasing osmotic potential. (**A**) The graphic shows the root growth rate for Col-0 and *ttl1* grown during 10 and 7 days in control and stressing −0.7 MPa and −1.2Mpa conditions, respectively. The magnitude of deceleration is higher in *ttl1*. Different letters indicate statistically significant differences (t Student *p* < 0.001; n = 30). (**B**) Images of the root apical meristem of Col-0 and *ttl1* mutant grown in control conditions. (**C**) Images of the root apical meristem of Col-0 and *ttl1* mutant after 7 days of growth in severe osmotic stress conditions (−1.2 MPa osmotic potential), showing the reduction in the number of cells in the proximal meristem (PM) and the reduction of the elongation zone (EZ) size.

Mutations in the Arabidopsis *TTL1* gene cause reduced tolerance to NaCl and osmotic stress [32]. The main phenotype caused by osmotic stress in *ttl1* mutants was swollen root tips and root growth arrest [31,32,33]; Figure 1C. In this work we aim to investigate the role of *TTL1* in maintaining primary root growth during osmotic stress adaptation. For this purpose, we grew Col-0 and *ttl1* seedlings in solid MS + 1.5% sucrose for the control condition and in solid MS + 1.5% sucrose containing 300 mM mannitol (corresponding to −0.7 MegaPascal (MPa) of osmotic potential) or 400 mM mannitol (corresponding to −1.2 MPa of osmotic potential). In control conditions, *ttl1* primary root growth rate was not significantly different from that of Col-0 (Figure 1A). Interestingly, root growth rate decelerates and is not arrested with increasing osmotic potential (Figure 1). Col-0 reduced 50% its root growth rate after growing during 7 days in a medium with −0.7 MPa of osmotic potential and 88% when exposed the same period to a medium with −1.2 MPa of osmotic potential. However, the root growth rate was significantly even more reduced in the *ttl1* mutant, 63% at −0.7 MPa and 95% at 1–2 MPa of osmotic potential (Figure 1A).

The root length is dependent on the balance between the rate of cell division and cell elongation. Usually, a high number of cells dividing at the RAM produces more cells that can elongate and differentiate, resulting in a high root growth rate [43]. Growth parameters affecting the root growth rate were determined in the cortical cell files from the stem cell initials near the quiescent center to the beginning of the differentiation zone in roots exposed to 7 days of osmotic stress [38,39].

**Figure 2:**
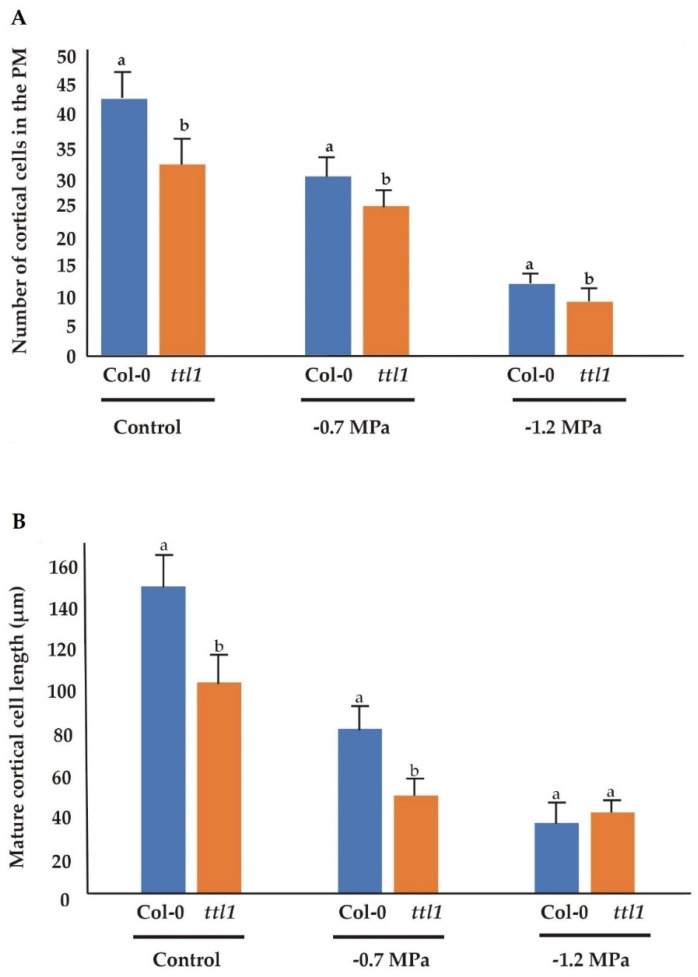
The number of cortical cells in the proximal meristem is reduced in *ttl1* mutant in correlation with the deceleration of root growth rate observed in osmotic stress condition. The figure shows the number of cortical cells in the proximal meristem (**A**) and the cortical mature cell length (**B**) for Col-0 and *ttl1* grown during 7 days in control, −0.7 MPa and −1.2 MPa osmotic stress conditions. Values are means ± standard deviation (SD). Different letters indicate statistically significant differences (*t* Student *p* < 0.05; *n* = 10). The experiments were repeated three times.

Although the number of cortical cells in the PM was reduced in both genotypes in response to increasing osmotic potential, the effect was different in Col-0 and *ttl1*. The number of cells in the PM in Col-0 was reduced by 30% after growing for 7 days at −0.7 MPa of osmotic potential and by 72% after 7 days at −1.2 MPa. However, the response of the *ttl1* mutant was more severe in both osmotic stress conditions: *ttl1* had a reduction of the cell number by 42% at −0.7 MPa and by 79% at −1.2 MPa compared to Col-0 PM in control conditions (Figure 2).

Osmotic stress also affected the length of mature cells in both genotypes. In the *ttl1* mutant −0.7 MPa caused a strong reduction in the length of mature cells, and a higher osmotic potential of −1.2 MPa reduced mature cell length an additional 7% (Figure 2). Interestingly, −0.7 MPa caused a more severe reduction effect on mature cell length than in the number of cortical cells in the PM compared to control conditions, 16% and 26% more in Col-0 and in *ttl1,* respectively. Moreover, at −1.2 MPa mature cell length was not significantly different between Col-0 and *ttl1* (Figure 2), indicating a mature length threshold to sustain root growth.

Furthermore, at −1.2 MPa, differences were also observed in the time dependence of cell number reduction in the PM. In Col-0 30% of the reduction occurred in the first 2 days and 65% after 7 days of exposure (Table S1). *ttl1*, however, reduced its PM cell number by 70% in the first 2 days of exposure (Tables S2 and S3) indicating that *TTL1* participate in the control of cell proliferation in the first days of stress adaptation.

The PM cell proliferation rate depends on the cells that are produced in the stem cell niche (SCN), the dividing cells at the PM, and the cells that transit to the EZ. We did not detect any morphological differences in the organization of the PM in *ttl1*, neither in control nor stress conditions. (Figure 1B). However, under osmotic stress the cells in *ttl1* transited more rapidly to the elongation zone and prematurely acquired characteristics of differentiated cells in the epidermis (i.e., root hairs; Figure 1C). In addition, osmotic stress produces a radial expansion in cells of the epidermis and cortex of the TZ and EZ in the *ttl1* mutant, an expansion that remains in differentiated cells (Figure 1C).

**Figure 3:**
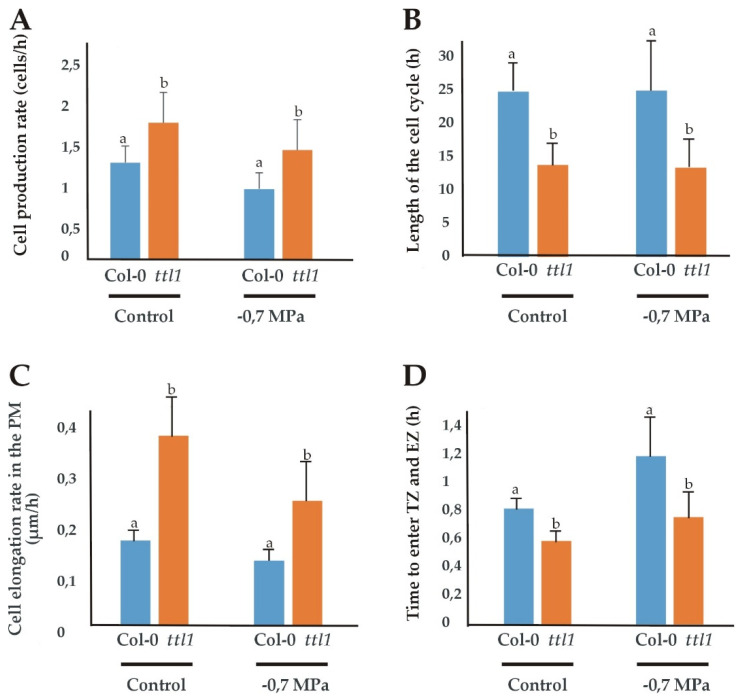
Growth parameters in the PM of Col-0 and *ttl1* roots grown in control and osmotic stress conditions. The figure shows the cell production rate (cells/h) (**A**); the length of the cell cycle (h) (**B**); the cell elongation rate (µm/h) (**C**) and the time to enter the transition zone (TZ) and elongation zone (EZ) (**D**) for Col-0 and *ttl1* grown during 7 days in control and −0.7 MPa osmotic stress conditions. Values are means ± standard deviation (SD). Different letters indicate statistically significant differences (*t* Student *p* < 0.05; *n* = 10).

A cortical cell profile analysis was used to estimate the cell production rate, the length of the cell cycle, the elongation rate in the PM and the average time between each cell entering the TZ and the EZ as described in Materials and Methods. The estimations were made with roots grown in control and −0.7 MPa of osmotic potential media because at −1.2 MPa the root growth rate was too low for obtaining reliable data (Figure 3).

Surprisingly, in control conditions, the *ttl1* mutant show a reduced number of cortical cells in the PM compared to Col-0 and *ttl1* cells at maturity did not elongate to the same extent as Col-0 cells (Figure 2). However, *ttl1* has a higher cell elongation rate in the PM than Col-0 (Figure 3C; Figure S5A) and the cells transit to the EZ faster than in Col-0 (Figure 3D). At −0.7 MPa of osmotic potential the cell number of the PM (Figure 1B and Figure 2), as well as the mature cell length were reduced in *ttl1*, explaining the root growth rate reduction in osmotic stress. The PM size reduction was due to a decrease in cell number in the PM and a premature transit towards the differentiation zone evidenced by epidermal cells closer to the QC acquiring differentiated characteristics (Figure 1C). The cell elongation rate estimated in the PM was reduced in the *ttl1* mutant indicating that the cells in the TZ did not reach a suitable size before transiting to the elongation zone under osmotic stress (Figure 3C).

### 3.2. Osmotic Stress Increases Cell Wall Stiffness of Root Elongation Zone Cells

**Figure 4:**
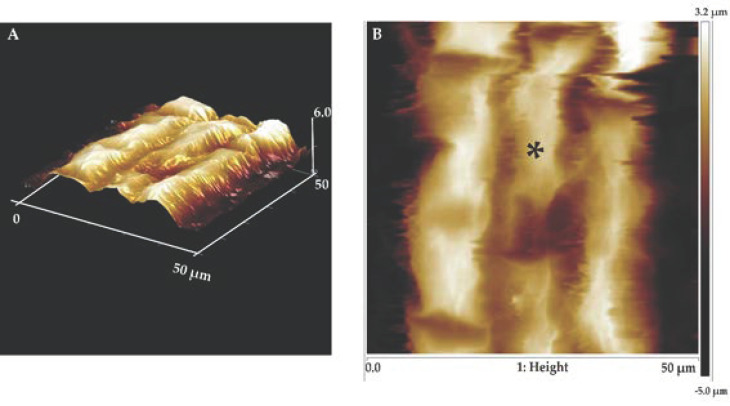
Atomic force microscopy (AFM) imaging of the surface of the root elongation zone cells at high resolution. Topography of a small area (50 × 50 µm) of the root elongation zone using contact mode in fluid. Friction image of a few cells of the root elongation zone of Col-0 (**A**); height images of the same zone of Col-0 indicating the location where force curves were obtained (**B**). * indicates the location where force curves were obtained.

Next, we studied the stiffness of epidermal cell walls in the root elongation zone of Col-0 and *ttl1* under control and osmotic stress using atomic force microscopy (Figure 4 and Table 1). The force curves on live cells of the root elongation zone showed lower indentation depth in Col-0 than in the *ttl1* mutant at an equally applied force (Figure S1A). A priori, these results indicate that Col-0 cell walls of epidermal cells in the elongation zone are stiffer. To confirm this, each curve was fitted by a second-order polynomial considering only the first 100 nm of each curve to avoid the effect of the substrate on the stiffness measurements. Figure S1B shows the Young’s modulus normalized histogram obtained from Col-0 (3 seedlings, 201 force curves) and *ttl1* (3 seedlings, 1184 force curves) samples. Each histogram was fitted with a Gaussian curve to obtain the mean and standard deviation of the Young’s modulus. The AEM of Col-0 (88.12 KPa ± 2.79 KPa) was significantly different (*p* < 0.05) from that of *ttl1* mutant (16.08 KPa ± 6.87 KPa). The mean of the elastic modulus was 448% higher for Col-0 than *ttl1* mutant (Table 1 and Table S4).

Under osmotic stress, the force curves on live cells also showed that the cell wall of Col-0 is stiffer than the cell wall of the *ttl1* mutant (Figure S2A). Figure S2B shows the Young’s modulus normalized histogram obtained from all Col-0 (3 seedlings, 263 force curves) and *ttl1* mutant (3 seedlings, 324 force curves) samples grown in osmotic stress. The AEM of Col-0 (164.98 KPa ± 2.66 KPa) was significantly different (*p* < 9.8971 × 10^−306^) from that of *ttl1* mutant (29.71 KPa ± 13.9 KPa). The mean of the elastic modulus was 455% higher for Col-0 than the *ttl1* mutant (Table 1 and Table S4). Interestingly, after 7 days of osmotic stress, we observed that the cell wall of epidermal cells of the elongation zone of both Col-0 and *ttl1* get 87% and 84% stiffer, respectively (Table 2 and Table S4).

To investigate whether the *ttl1* stiffness-defective phenotype could be related to defects in cell wall structural composition, we analyze the mechanical properties of the cell walls of EZ cells of *prc1-1,* a CESA6-null mutant that present a 50% reduction in the content of crystalline cellulose [35]. Even though the AEM of *ttl1* was statistically different from the AEM of *prc1-1*, it was in the same order of magnitude in control conditions (Table 1 and Table S4). For both mutants cell walls of epidermal cells in the elongation zone stiffen in response to osmotic stress (Table 2 and Table S4). However, neither reaches the order of magnitude of Col-0, explaining the swelling phenotype observed in this condition (Table 1 and Table 2; Table S4). As *prc1-1* is known to have less cellulose crystalline in its cell walls [35] our data suggests that *ttl1* may have a different composition or organization of its cell wall compared to Col-0.

We calculated the level of epistasis by comparing the AEM *ttl1prc1-1* value of the double mutant with the product of the AEM *ttl1* and AEM *prc1-1* values of the corresponding single mutants. The epistasis mean value for the *ttl1prc1-1* double mutant grown in control conditions was 0.2 ± 0.1. Epistatic interactions help to elucidate functional association between genes; in this case, the values obtained suggest that *TTL1* and *CESA6* could participate in a sequential manner in the process of cell wall formation [42].

The *ttl1prc1-1* double mutant had a slower root growth rate than Col-0 and *ttl1* in control conditions (t Student *p* < 0.001; *n* = 30; Figure S3). In this condition the number of cells in the PM of the double mutant (27 ± 2) was significantly different (Student’s *t*-test *p* < 0.05) from Col-0 (43 ± 5) and *ttl1* (33 ± 5) but did not differentiate from *prc1-1* (27 ± 2). The length that cells reached at maturity was significantly shorter in the double mutant in comparison to *ttl1*, *prc1-1* and Col-0 (Table S5). Due to the extreme phenotype of *ttl1prc1-1* grown for 7 days in −1.2 MPa we were unable to obtain force curves that allowed us the proper analysis (Figure S4).

The root growth rate in control conditions of the *ttl1prc1-1* is significantly lower than Col-0, *ttl1* and *prc1-1* (Figure S3). Additionally, the root growth rate decelerated more in the *ttl1prc1-1* double mutants than in the single mutants at increasing osmotic potential. Interestingly, at −0.7 MPa, both the cell number at the PM and the mature cell length were significantly reduced in *ttl1prc1-1* compared to Col-0, *ttl1* and *prc1-1* (Table S6). At −1.2 MPa there was a significant reduction in the number of cells in the PM of *ttl1prc1-1*, but there were no differences in the mature cell length with Col-0 *ttl1* and *prc1-1* (Table S7).

### 3.3. Cell-Specific Root Expression Analysis of TTL1 Using Transgenic pTTL1::GUS Plants

**Figure 5:**
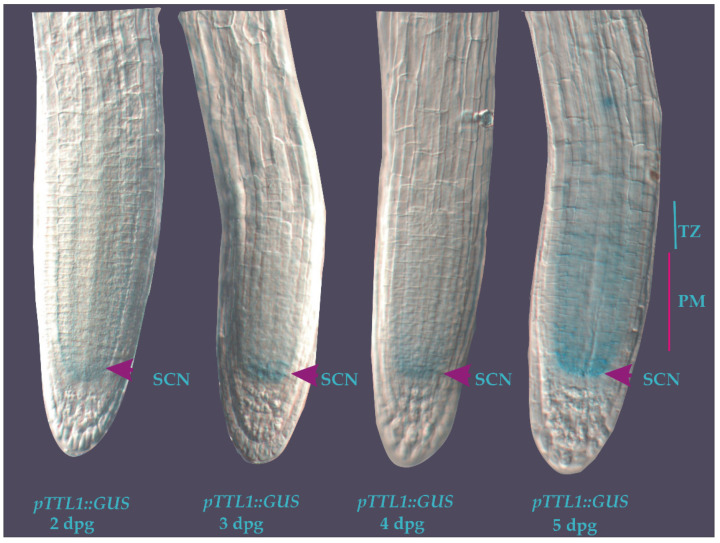
Expression patterns of *TTL1* in Arabidopsis primary root using *promoter::GUS* analysis from 2 to 5 days post germination in control medium. *pTTL1::GUS* roots showing GUS signal in the stem cell niche (SCN) from 2 to 4 dpg (arrowheads). At 5 dpg the signal is also localized in the cortex, endodermis, and stela cells in the PM and transition zone (TZ). *n* = 15.

In order to get further insight into the role of *TTL1*, we used the *pTTL1::GUS* transcriptional line previously reported [32]. We first analyzed the expression pattern of *TTL1* during primary root development. Specific GUS staining was observed at the QC since the second day post-germination until day 4. At day 5 post-germination, GUS staining spread towards cells of the cortex, endodermis, and stele in the PM and TZ (Figure 5). These observations are consistent with the expression pattern reported in the publicly available microarray data present in the Electronic Fluorescent Pictograph browser [44] for roots spatiotemporal data [45] in which the highest levels of *TTL1* expression were found in the QC (134.83 ± 30.83) and the epidermis and columella lateral (183.33 ± 11.93), followed by columella cells (82.61 ± 23.1), cortex (75.72 ± 7.76) and stele (68.45 ± 21.83). The expression values are mean and standard deviation of ATH1 normalized data by GCOS method, TGT value of 100. Values are average of two to three replicates, with spatiotemporal expression levels imputed by an EM algorithm [46]

### 3.4. TTL1 Expression Analysis in the Primary Root in Response to Hyperosmotic Stress and Hormonal Regulation

**Figure 6:**
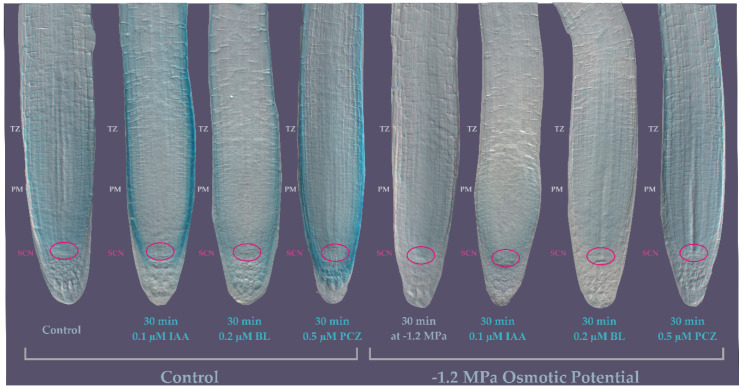
Expression patterns of *TTL1* Arabidopsis primary root using *promoter::GUS* analysis in response to osmotic potential and hormones. *pTTL1::GUS* roots grown in control conditions showing GUS signal localized to the stem cell niche (SCN) and the proximal meristem (PM) epidermis, cortex, endodermis and stele. *pTTL1::GUS* roots exposed during 30 min to control medium supplemented with 0.1 µM IAA showing GUS signal localized in the epidermis. *pTTL1::GUS* roots exposed during 30 min to control medium supplemented with 0.2 µM BL showing GUS signal in lateral root cap and epidermal cells. *pTTL1::GUS* roots exposed during 30 min to control medium supplemented with 0.5 µM PCZ showing GUS signal in columella, lateral root cap and epidermal cells. *pTTL1::GUS* roots grown in −1.2 MPa osmotic potential medium showing almost no GUS signal in the SCN and PM. *pTTL1::GUS* roots exposed during 30′ to −1.2 MPa osmotic potential medium supplemented with 0.1 µM IAA showing a pale signal in epidermal and cortex cells in the PM. *pTTL1::GUS* roots exposed during 30 min to −1.2 MPa osmotic potential medium supplemented with 0.2 µM BL showing a pale GUS signal in the PM. *pTTL1::GUS* roots exposed during 30 min to −1.2 MPa osmotic potential medium supplemented with 0.5 µM PCZ showing a pale GUS signal distributed similar to the one observed in control conditions without hormones supplemented. *n* = 15.

Next, we investigated the expression of *TTL1* in response to indole acetic acid (IAA), epibrassinolide (BL) and the BR biosynthesis inhibitor propiconazole (PCZ) in the roots of 7 dpg *pTTL1::GUS* seedlings growing in control and under osmotic stress. The GUS signal in 7 dpg seedlings grown in control media was localized to the SCN, PM, TZ and EZ. In the PM the signal was more intense in epidermis, cortex and endodermis in comparison to the stele. At the TZ and EZ a paler signal was observed (Figure 6). Roots of *pTTL1::GUS* seedlings exposed for 30 min in control media supplemented with 0.1 µM IAA increased the GUS signal in epidermal cells compared to roots in medium without IAA (Figure 6). *pTTL1::GUS* roots exposed for 30 min to 0.2 µM BL showed the GUS signal in the lateral root cap and epidermal cells (Figure 6). Finally, in *pTTL1::GUS* roots grown in presence of 0.5 µM PCZ GUS signal was localized in the columella, lateral root cap and epidermis (Figure 6). Interestingly, both IAA and PCZ treatments increased the GUS signal in the epidermis at a similar level. Thirty minutes of osmotic stress caused a severe and transient reduction of the GUS signal in the roots of *pTTL1::GUS* seedlings (Figure 6) that re-appear after 1 h of stress treatment (Figure S6).

The only treatment able to restore GUS signal to the localization and intensity of the control condition under osmotic stress was the treatment with 0.5 µM PCZ (Figure 6).

### 3.5. DR5::GUS Signal Localization in the Quiescent Center (QC) Did Not Change during the Exposure to Severe Osmotic Stress

Next, we determined whether the changes in *ttl1* root growth observed during osmotic stress were related to the known mechanisms regulating root growth. First we investigated the auxin gradients in *ttl1* using the well-stablished *DR5::GUS* marker [47].

**Figure 7:**
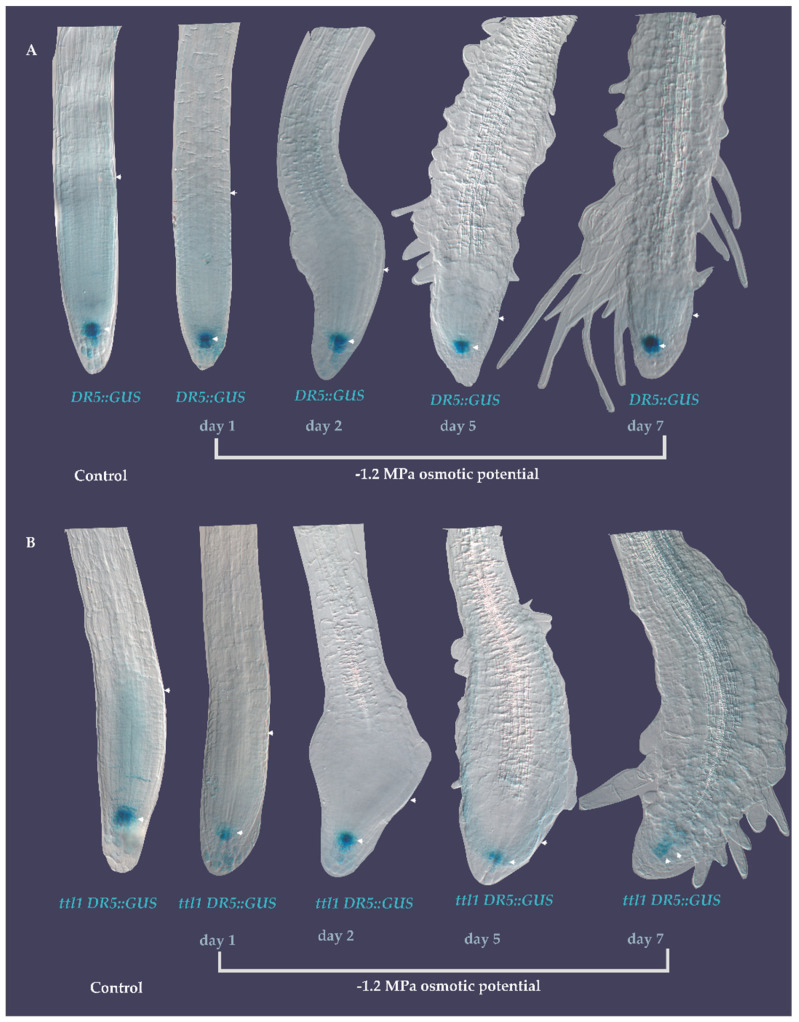
Expression pattern of auxin in response to 7 days of severe osmotic stress. (**A**) *DR5::GUS* in Col-0 in control medium and during 7 days of severe osmotic stress showing localization in the QC. (**B**) *DR5::GUS* in *ttl1* mutant showing the same localization as in Col-0. Arrowheads indicate the size of the PM (from the QC to the TZ).

For this, we introduced the *DR5::GUS* marker into the *ttl1* by crossing. As shown in Figure 7A,B, *DR5::GUS* had a similar expression pattern in Col-0 and *ttl1* background. However, monitoring the roots of *DR5::GUS seedlings* during 7 days of severe osmotic stress showed that there was a significant decrease of the signal in the *ttl1* mutant (Figure 7B) compared to Col-0 (Figure 7A) suggesting that *TTL1* could mediate part of the components of root growth through auxin response during osmotic stress.

### 3.6. Osmotic Stress Suppresses Brassinosteroids (BR) Signaling in Epidermal Cells of Elongation Zone (EZ)

**Figure 8:**
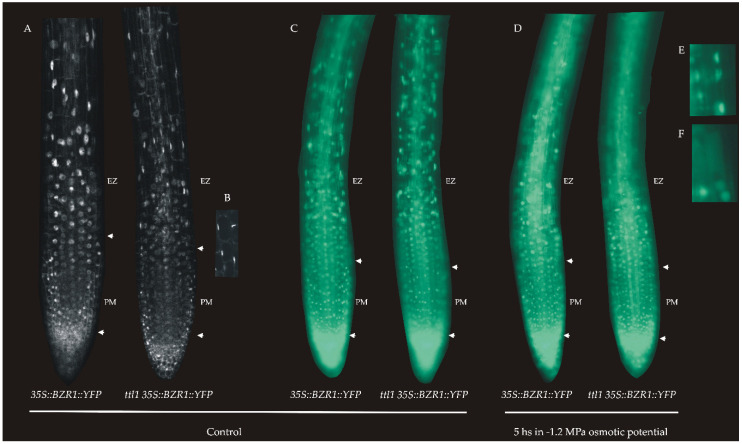
(**A**) Expression pattern of BZR1 in roots of Col-0 and *ttl1* mutant grown during 7 days in control conditions. (**B**) Zoomed in view of TZ showing the nuclear expression pattern in *ttl1*. This pattern suggests that cellular expansion begins earlier than in Col-0. Images were taken with Confocal Microscopy Zeiss LSM800—AiryScan *n* = 10. (**C**–**F**). Expression pattern of brassinosteroids (BR) in response to 5 h of severe osmotic stress. (**C**) *35S::BZR1::YFP* in Col-0 and *ttl1* mutant plants grown in control medium showing the YFP signal in the nuclei of EZ epidermal cells. (**D**) *35S::BZR1::YFP* in Col-0 and *ttl1* mutant plants grown during 5 h of severe osmotic stress showing YFP signal localized in the cytoplasm of epidermal EZ cells. (**E**) Zoomed in view of signal in epidermal EZ cells of *ttl1* mutant grown in control condition showing the nuclei localization of YFP signal. (**F**) Zoomed in view of signal in epidermal EZ cells of *ttl1* mutant grown in hyperosmotic condition showing the cytoplasmic localization of YFP signal. Images were taken with Epifluorescence microscope ZEIZZ—AXIO Imager. M2.

It has been reported that BZR1 has a specific pattern of expression in the epidermal cells of the root apical meristem and that the level of nuclear BZR1 provides a readout of BR signaling [17]. BZR1-YFP fusion protein driven by a constitutive 35S promoter accumulated at a low level in the nuclei of cells in SCN and at a high level in the nuclei of epidermal cells in TZ and EZ in correlation with rapid cell elongation [17]. In order to analyze if the BR signaling is affected in the *ttl1* mutant during osmotic stress we crossed *ttl1* with *35S::BZR1::YFP* and selected a *ttl1* carrying the *35S::BZR1::YFP* marker. The expression of *35S::BZR1::YFP* in the roots of *ttl1* seedlings grown in control conditions showed a similar pattern to that of Col-0, i.e., nuclear localization increased as cells enters the TZ-EZ (Figure 8A). We observed that the cells of the TZ of the *ttl1* mutant showed a change in the characteristic shape of the nuclear signal (zoom in Figure 8B), where the nucleus seemed to be compressed to the sides of the cells which suggests that the cellular expansion begins earlier in the *ttl1* mutant. In response to 5 h of severe osmotic stress (−1.2 MPa of osmotic potential) BZR1 pattern localized mostly in the cytoplasm in the EZ epidermal cells in both Col-0 and *ttl1* mutant (Figure 8D–F). However, this pattern begins in cells closer to the PM in the *ttl1* mutant than in Col-0 (Figure 8D–F). This pattern of BZR1 in response to osmotic stress agreed with the observation that Col-0 mature cells experienced a 46% of length reduction during severe osmotic stress compared to the control condition and the *ttl1* mutant mature cells a 73% of length reduction during severe osmotic stress compared to Col-0 in control conditions (Figure 2). These observations suggested that the reduction in elongation in EZ cells during osmotic stress can partially be explained by the BR signaling pathway.

### 3.7. Exogenous BR Application Did Not Recover the Root Length Reduction of ttl1 Grown under Osmotic Stress

BR at nM to µM concentrations have inhibitory effects on primary root growth [48]. Moreover, the use of PCZ also produce a reduction in primary root growth [49]. The finding that the homologous TTL3 protein interacts in vivo with most BR signaling components [33] makes us evaluate a possible role of *TTL1* in BR signaling. It was previously described that *ttl1*, similarly to *ttl3* and *ttl4*, presented a decreased sensitivity to BL when seedling grown for 4 days in half strength MS medium were transferred to the same medium supplemented with 0.1 µM of BL, and root length was measured 6 days later [33]. To further investigate the effect of BR inhibiting concentrations on primary root length, we tested *ttl1* root elongation in our phenotypic analysis experimental conditions. After 5 days of growth in control medium, plus 9 days of growth in control medium supplemented with 0.2 µM of BL, Col-0 root length was 72% compared to control conditions (Table 3 and Table S8). However, the *ttl1* mutant showed more sensitivity to BL, since the root length of *ttl1* grown in control medium supplemented with 0.1 µM of BL was 66% compared to the root length of Col-0 grown in control conditions and 58% when grown in control medium supplemented with 0.2 µM of BL (Table 3 and Table S8). This result supports the possibility that *ttl1* is defective in BR signaling and also suggests that the inhibition of root growth by BL in *ttl1* may be less or greater than Col-0 depending on the age of the seedlings and/or the duration of the BR treatment. The upregulation of BR biosynthetic genes has been reported for BR signaling mutants such as *bri1-5* [50], *bri-301* [51], and *bik1* [52] and is caused by a lack of feedback regulation in the expression of these biosynthetic genes [53,54,55]. It is possible that BR biosynthetic genes are induced in *ttl1* after large periods (9 days) of BL treatment, overcompensating its initial hyposensitivity to BR.

In addition, we studied the effect of PCZ in the root length. After 9 days of growth in control medium supplemented with PCZ we did not observe significant differences in Col-0 root length compared with Col-0 roots grown in control medium (Table 3 and Table S8), but the root length of *ttl1* was 67% compared to Col-0 root length grown in control conditions (Table 3 and Table S8). The response of *ttl1* was similar to the observed in *bri1-5* suggesting that *ttl1* responds to changes in BR homeostasis [49].

Furthermore, we evaluated the root length in hyperosmotic media and hyperosmotic media supplemented with BL or PCZ (Table 3 and Table S8). Interestingly, growth during 10 days in control medium supplemented with BL produced a reduction in the length of the root of *ttl1* mutant as severe as 9 days growing in severe osmotic stress (Table 3 and Table S8). The combination of 400 mM Mannitol + 0.2 µM of BL produced Col-0 roots 20% longer than in 400 mM de Mannitol. On the contrary, BL did not produce significant changes in the length of *ttl1* roots growing in osmotic stress (Table 3 and Table S8). We did not find a significant effect on root length of both genotypes of PCZ in osmotic stress conditions (Table 3 and Table S8).

In summary, the effect of recuperation of the root length of BL treatment observed in Col-0 roots growing in hyperosmotic medium that was not observed in the *ttl1* mutant suggests a possible connection between *TTL1*, BR and root growth osmotic stress adaptation.

## 4. Discussion

Water availability is a factor that strongly limits plant growth and its uptake by the roots is affected by many abiotic stresses such as drought [12,21,56,57,58]. Roots sense drought through specific cell types such as stem, cortical and vascular cells and respond adaptively to avoid dehydration, [4,59,60,61,62,63]. Auxins modulates root elongation in the RAM [64], abscisic acid (ABA) participate in EZ cortex cells sensing water potential [65] and vascular BRL3 overexpression confers drought resistance [63].

TTL proteins comprise a novel family of proteins that are specific to plants that appeared in the transition from aquatic environments to land, hinting at their potential role in plant drought-stress adaptation during terrestrial colonization [31,32,66]. Mutations in the Arabidopsis *TTL1* gene cause reduced tolerance to osmotic stress evidenced by an arrest in root growth and root swelling, which makes it an interesting model for exploring how root growth is regulated under osmotic stress conditions [31,32,33]. In this work we evaluated primary root growth of the *ttl1* mutant during 7 days of growth comparing two osmotic potentials (−0.7 MPa and −1.2 MPa) generated with mannitol. Interestingly, *ttl1* root growth rate decelerates with increasing osmotic potential and this reduction is significantly greater than the Col-0 root growth rate reduction. During osmotic stress, the reduction in primary root growth rate could be explained by the inhibition in cell elongation in EZ (measured by the reduction in mature cell length) followed by the reduction in the number of cortical cells in the PM. Similar results were obtained in response to saline and osmotic stress in Arabidopsis [15,20,23]. At −0.7 MPa osmotic potential mature cell length was more affected than the cell number at the PM, whereas at −1.2M Pa both parameters were strongly affected. This points out that the severity of the stress affects each domain of the RAM differently, as described before for the meristematic zone and the transition zone of the root [23]. In this study, at −0.7 MPa of osmotic potential we observed a premature cell transit towards the transition and elongation zone and to the differentiation zone coincident with the observation of epidermal cells closer to the QC acquiring differentiated characteristics as previously described for Col-0 accession growing in hyperosmotic conditions [23].

Cellular expansion plays an important role during root growth and development. In cells that expand longitudinally, cellulose microfibrils are deposited perpendicular to the expansion axis, restricting radial expansion [25,43]. The fact that cellulose deficient mutants have swelling phenotypes and slower root growth rates, indicate that at the cellular level, the size, the expansion direction [10] and the mechanical properties of the cell wall itself contribute to the signaling process of root growth arrest [22,57]. In this work, we observed that mature cell length was reduced with the severity of the osmotic stress, at −0.7 MPa mature cell length was significantly different between Col-0 and *ttl1*; at −1.2 MPa, however, mature cell length was not statistically different between genotypes. These data suggest the existence of a minimal cell length threshold, and/ or that the physical properties of the cell wall contribute to the signaling process to sustain root growth rate in osmotic stress.

We observed that the *ttl1* root swelling phenotype was enhanced at severe osmotic stress condition (−1.2MPa) and appeared after the second day of growth in osmotic stress affecting epidermis and cortex cells in the TZ. Interestingly, it was also present in Col-0, however in this case as the time of exposure to stress progresses the cells in the TZ of Col-0 adapted and the swelling phenotype disappeared. Some authors reported that Col-0 epidermal and cortex cells swell when the roots were subjected to a hyperosmotic shock (−1.2 MPa) [23], or to saline stress [67], and this has also been reported in plants with altered cell wall biogenesis [24,34,35,68,69]. The swelling phenotype is determined by the physicochemical properties of the cell wall [21,22,27]. The TZ domain is characterized by the cell wall structure alterations and vacuolization preparing the cells for the rapid elongation in the EZ which makes the TZ more sensitive to the osmotic perception and suitable to develop the swelling phenotype [20].

To examine whether osmotic stress and *TTL1* affect the cell wall’s mechanical properties at the EZ, we used atomic force microscopy (AFM). Unexpectedly, in control conditions, the elastic modulus’ mean was 448% higher for Col-0 than *ttl1* mutant, meaning that *ttl1* has its cell walls more elastic. Moreover, after 7 days of severe osmotic stress (−1.2 MPa), we found that the cell wall’s stiffness in the EZ increases. Even though, both genotypes behave in this way, the increase in stiffness observed in the cell walls of the EZ cells of the *ttl1* mutant did not reach the magnitude of the stiffness of Col-0 cell walls, explaining why the root swelling phenotype persists in the mutant.

Analysis of the mechanical properties of the cell walls of EZ cells of *prc1-1* mutant indicated that the cell walls of epidermal cells in the EZ of *prc1-1* were more elastic than Col-0, as was already reported for all the *cesa* mutants in the shoot apical meristem (SAM) [70]. This agreed with the reduced content of crystalline cellulose of *prc1-1* [35]. Moreover, the root growth rate and AEM of the *ttl1prc1-1* double mutant showed that *TTL1* genetically interacts with *CESA6*.

Feng et al., 2018 reported that after salt treatment, the cell walls of the epidermal cells in root EZ of wild-type Arabidopsis has an overall drop in wall stiffness, which they recovered at the beginning of the growth recovery phase. However, mutants with impaired cell wall integrity as *fer* failed to restore cell wall stiffness. In our conditions we did not see a drop in wall stiffness after osmotic stress, showing that osmotic stress induced by NaCl include also an ionic stress, supporting the hypothesis that sodium ions could directly disrupt load-bearing ionic interactions with the wall [22].

Auxin and BR have been proved to act antagonistically in the process of cell elongation in the root. Transcriptomic analysis in wild type plants showed that BR-induced but auxin-repressed genes tend to be related to cell-wall organization and biogenesis and water transport, whereas BR-repressed but auxin-induced genes tend to be involved in ribosome biogenesis [17]. At the cellular level, auxin treatment to the wild-type plants reduced cell elongation and increased lateral cell expansion in the meristem zone and radial dimension of root tip, in contrast to BR. Furthermore, auxin treatment to the wild-type plants dramatically inhibited cell elongation in the elongation zone [17]. The wild-type roots treated with BL showed early initiation of elongation in the transition zone, whereas IAA showed similar initiation of the elongation as the mock but failed to elongate further in the elongation zone compared to BL-treated roots and the mock condition [17]. Furthermore, reaching a critical cell size is affected by BR controlling the expansion process through cell wall modification, leading to a normal progression of the cell cycle, and contributing to the establishment of the size of the root meristem. *BRI1* activity in the epidermis initiates a signal that regulates gene expression of the inner meristematic cell files [14].

When we investigated the role of *TTL1* focusing on auxin and BR signaling the auxin response pattern did not change in the *ttl1* mutant during hyperosmotic stress, thus the *DR5::GUS* signal localized to the QC as in Col-0, although its intensity diminished as the treatment with osmotic stress advanced. In addition, 5 h of hyperosmotic stress was enough to delocalize BZR1 from the nucleus of epidermal cells of the root EZ, in both genotypes, suggesting that osmotic stress also dynamically represses the BR signaling pathway as was mentioned for saline stress [15]. These observations concur with the fact that mature cell length was more affected by osmotic stress in *ttl1* than the number of cells in the PM.

Interestingly, *ttl1* has a reduced number of cortical cells in the PM compared to Col-0 that did not elongate to the same extent as Col-0 cells. A reduced PM size and mature cell length is also observed in the BR-deficient mutant *dwf4* and the BR insensitive mutant *bri1* [13,14,17,39]. The BZR1 pattern confirmed that the elongation rate of PM cortical cells is faster in *ttl1* mutant in control conditions, resembling what is observed when Col-0 roots are treated with BL [17]. However, *ttl1* mature cell size in control conditions, shorter than in Col-0, resembled more the effect of the combination of auxin and BR treatment to Col-0 [17]. Our study revealed that *pTTL1::GUS* respond to auxins, BR, PCZ increasing the intensity of the signal in the epidermis of both meristematic zones. It was previously described that *ttl1* presented a decreased sensitivity to BL [33]. In this study, *ttl1* showed more sensitivity to BL than Col-0, supporting the notion that *ttl1* is defective in BR signaling and suggests that the inhibition of root growth by BL in *ttl1* may be less or greater than Col-0 depending on the age of the seedlings and/or the duration of the BR treatment. The upregulation of BR biosynthetic genes has been reported for BR signaling mutants such as *bri1-5* [50], *bri1-301* [51], and *bik1* [52] and is caused by a lack of feedback regulation in the expression of these biosynthetic genes [53,54,55]. It is possible that the BR biosynthetic gene is induced in *ttl1* after large periods of BL treatment, overcompensating its initial hyposensitivity observed to BR. Whether all the observed changes are direct effects of BR homeostasis or emerge indirectly, for example, from hormonal crosstalk with auxin, remains to be determined.

*pTTL1::GUS* signal was visible in the QC from day 2 to day 5 after germination and the *ttl1* mutant has less cortical cells at the PM at day 7 after germination, further informing the possible function of this gene during PM size determination. In control conditions, 7 days after germination the *pTTL1::GUS* signal appeared in the epidermis and the cortex of the PM and TZ. In contrast, 30 min of hyperosmotic stress reduced the *pTTL1::GUS* signal and only PCZ restored *pTTL1::GUS* signal to the intensity of control conditions.

## 5. Conclusions

In summary, in this work we used atomic force microscopy to identify a role of *TTL1* in the control of cell wall integrity during root anisotropic growth and found a genetic interaction between *TTL1* and *CESA6*. In addition, we found that during osmotic stress, anisotropic growth is the first limiting process to establish root growth rate adaptation.

**Figure 9:**
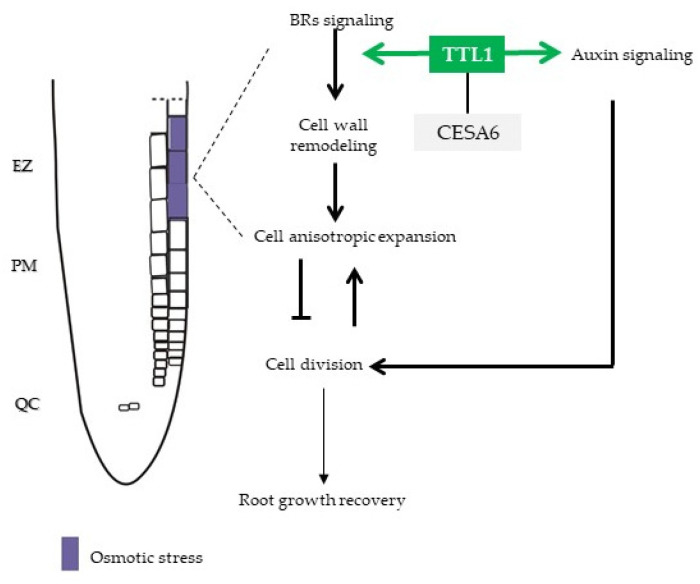
Model that illustrates a scenario for primary root growth recovery during osmotic stress. Osmotic stress is perceived in epidermal cells of EZ and integrates through *TTL1* in the BR/auxin signaling pathway that regulates balanced root growth. *TTL1* maintain cell wall integrity to sustain cell anisotropic expansion necessary to continuous primary root growth. EZ: Elongation Zone; PM: Proximal Meristem; QC: Quiescent Center; BR: brassinosteroid.

Moreover, the temporary suppression of *TTL1* expression and BR pathway in response to osmotic stress and the response to BR treatment together with the evidence of *in vivo* interaction of TTL3 with most of the BR signaling components [33] suggests a role for *TTL1* mediating root growth adaptation to osmotic stress through auxin/BR homeostasis (Figure 9).

## Figures and Tables

**Table 1:** Cell walls apparent elastic modulus measured in cells of the elongation zone of roots of Col-0, *ttl1, prc1-1 and ttl1prc1-1* grown in control conditions.

Control	Y (kPa)	N	Error
Col-0	88.12 ± 2.79 a	201	0.19
*ttl1*	16.08 ± 6.87 b	1184	0.19
*prc1-1*	18.08 ± 8.04 c	2231	0.17
*ttl1 prc1-1*	27.06 ± 8.5 d	1052	0.26

Values are means ± SD; 200-2231 force curves were obtained from the central part of elongation cells of 3 roots for each growing condition. Seedlings were attached to petri dishes with silicone and covered with 1× phosphate-buffered saline (PBS) solution. Normalized Young’s modulus histograms were obtained from all experimental conditions. Each histogram was fitted with a Gaussian curve. Statistical significance is as follows: *p* < 0.05 (by Student’s *t*-test) for a vs. b and *p* < 9.8971 × 10^−306^ (by Student’s *t*-test) for c vs. d.

**Table 2:** Cell walls apparent elastic modulus measured in cells of the elongation zone of roots of Col-0, *ttl1, prc1-1 and ttl1prc1-1* grown in osmotic stress conditions (−1.2 MPa).

Osmotic Stress (−1.2 MPa)	Y (kPa)	N	Error
Col-0	164.98 ± 2.66 a	263	0.16
*ttl1*	29.71 ± 13.9 b	324	0.77
*prc1-1*	51.54 ± 24.86 c	1161	0.72

Values are means ± SD; 200–600 force curves were obtained from the central part of elongation cells of 3 roots for each growing condition. Seedlings were attached to petri dishes with silicone and covered with 1× PBS phosphate-buffered saline (PBS) solution. Normalized Young’s modulus histograms were obtained from all experimental conditions. Each histogram was fitted with a Gaussian curve. Statistical significance is as follows: *p* < 0.05 (by Student’s *t*-test).

**Table 3:**
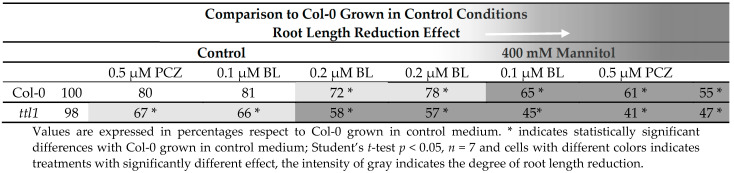
Different size of letters between header and footer, please correct.

## Data Availability

The data presented in this study are available in the article or supplementary materials.

## Supplementary Materials

The following are available online at https://www.mdpi.com/2073-4425/12/2/236/s1. Table S1: Time dependence of Col-0 primary root response to osmotic stress. Table S2: Time dependence of *ttl1* primary root response to osmotic stress. Table S3: Time dependence of Col-0 and *ttl1* primary root response to osmotic stress. Table S4: *p* values for *t*-Test comparison of AEM in Col-0, *ttl1*, *prc1-1*, *ttl1 prc1-1* grown in control and −1.2 MPa osmotic potential for data in Table 1 and Table 2 of the main text. Table S5: Cortical cell number in the PM and Mature cortical cell length meassured in control conditions. Table S6: Cortical cell number in the PM and Mature cortical cell length meassured at −0.7 MPa. Table S7: Cortical cell number in the PM and Mature cortical cell length meassured at −1.2 MPa. Table S8: Root length of Col-0 and *ttl1* after 9 days of growth in medium with, 0.2 µM of BL, 0.5 µM of PCZ, without or with supplementation of 400 mM. Figure S1: Force curves and apparent elastic modulus (AEM) of cell walls of epidermal cells in the EZ of Col-0 and *ttl1* roots grown in control conditions. Figure S2: Force curves and apparent elastic modulus (AEM) of cell walls of epidermal cells in the EZ of Col-0 and *ttl1* roots grown under osmotic stress. Figure S3: The *ttl1prc1-1* mutant exhibits greater reduction of root growth rate than Col-0 in response to increasing osmotic potential. Figure S4: Histogram of Young’s modulus obtained from force curves on 9 different cells of three different plants of the *ttl1prc1-1* double mutant. Figure S5: Cell size in the PM and EZ determined in control conditions for Col-0 and *ttl1* mutant. Figure S6: *pTTL1::GUS* signal in response to different time of osmotic stress (−1.2 MPa).

## Abbreviations

RAM	root apical meristem
SCN	stem cell niche
QC	quiescent center
PM	proximal meristem
EZ	elongation zone
TZ	transition zone
DZ	differentiation zone
GFP	green fluorescent protein
YFP	yellow fluorescent protein
GUS	β-glucuronidase
MPa	MegaPascal
NaCl	sodium chloride
ABA	abscisic acid
BR	brassinosteroid
BL	epibrasinolide
PCZ	propiconazole
AFM	atomic force microscopy
AEM	Young’s Modulus
