# Supplementary material for: The Arabidopsis TETRATRICOPEPTIDE THIOREDOXIN-LIKE 1 Gene Is Involved in Anisotropic Root Growth during Osmotic Stress Adaptation
Source: Genes (Basel). 2021 Feb 7;12(2):236. doi: 10.3390/genes12020236 (PMC7915054; doi:10.3390/genes12020236)
Supplement: Supplementary file 1 [file genes-12-00236-s001.pdf]

genes

MDPI

Supplementary Materials

Table S 1. Time dependence of Col-0 primary root response to osmotic stress. The table shows the effect of the exposition time to 400 mM mannitol on PM parameters of Col-0. Values are expressed as percentage relative to the wild type grown in control conditions. Values with the same number of * are not statistically different; Tukey, p>0.05. n= 20.

Effect of exposition time to 400mM mannitol on PM	**Control**	**2 days in 400 mM mannitol**	**7 days in 400 mM mannitol**
PM	**Col-0**	**Col-0**	**Col-0**
N° cortical cells	100%*	77,2%**	40,4%***
EZ			
Length of EZ	100%*	56%*	15,5%**
Width of EZ	100%*	108,6%*	88,5%*

Table S 2. Time dependence of *ttl1* primary root response to osmotic stress. The table shows the effect of the exposition time to 400 mM mannitol on PM parameters of *ttl1*. Values are expressed as percentage relative to the wild type grown in control conditions. Values with the same number of * are not statistically different; Tukey, p>0.05. n= 20.

Effect of exposition time to 400mM mannitol on PM	**Control**	**2 days in 400 mM mannitol**	**7 days in 400 mM mannitol**
PM	*ttl1*	*ttl1*	*ttl1*
N° cortical cells	100%*	36,6%**	37%**
EZ			
Length of EZ	100%*	15,9%**	9,8%**
Width of EZ	100%*	132,8%**	87,6%***

Table S 3. Time dependence of Col-0 and *ttl1* primary root response to osmotic stress. The table shows the effect of the exposition time to 400 mM mannitol on PM parameters of *ttl1*. Comparisons were made between days of osmotic stress exposition for each genotype. Values with the same letter are not statistically different; Tukey, p>0.05. n= 20.

Effect of exposition time to 400mM mannitol on PM	**Control**		**2 days in 400 mM mannitol**		**7 days in 400 mM mannitol**	
PM	**Col-0**	***ttl1***	**Col-0**	***ttl1***	**Col-0**	***ttl1***
N° cortical cells	29 ± 3**a**	23 ± 5**a**	22 ± 0.58**b**	8 ± 1**b**	12 ± 0,58**c**	8 ± 1**b**
EZ						
Length of EZ (μm)	578.35 ± 185.8**a**	545.9 ± 147.3**a**	312 ± 173**ab**	95.4 ± 18.7**b**	86.7 ± 40**b**	58.6 ± 10**b**
Width of EZ (μm)	111.2 ± 3.8**a**	132.3 ± 9.5**a**	120.7 ± 24.6**a**	175.7 ± 13.5**b**	98.3 ± 11.9**a**	115.9 ± 11.3**c**

Table S 4. *p* values for t-Test comparison of AEM in Col-0, *ttl1*, *prc1-1*, *ttl1 prc1-1* grown in control and -1.2 MPa osmotic potential for data in Table 1 and Table 2 of the main text.

	Col-0 Control	Col-0 -1.2 MPa	*prc1-1 Control*	*prc1-1* -1.2 MPa	*ttl1*	*ttl1* -1.2 MPa	*ttl1prc1-1* Control	*ttl1prc1-1* -1.2 MPa
Col-0 Control	X	8.37 x 10 ^ -263	5.03 x 10 ^ -176	4.81 x 10 ^ -53	1.29 x 10 ^ -206	3.84x10^-126	1.05x10^-164	1.07x10^-181
Col-0 -1.2 MPa		x	0	9.27 x 10 ^ -181	0	3.79 x 10 ^ -259	9.27 x 10 ^ -181	9.27 x 10 ^ -181
prc1-1 Control			x	8.3030e-239	1.0033e-24	3.3889e-12	2.2043e-66	1.2089e-07
prc1-1 -1.2 MPa				x	9.6035e-269	1.3365e-34	1.1365e-148	3.3156e-53
ttl1 Control					x	4.2909e-32	5.3264e-159	0.2022
ttl1 -1.2 MPa						x	5.2489e-06	2.8942e-19
ttl1prc1-1 Control							x	2.1581e-12
ttl1prc1-1 -1.2 MPa								x

Table S 5. Cortical cell number in the PM and Mature cortical cell length meassured in control conditions. The tables show the number of cortical cells in the proximal meristem and the cortical mature cell length for Col-0, *ttl1, prc1-1 and ttl1prc1-1* grown during 7 days in control conditions. Values are mean and SD. Different letters indicates statistically significant differences; Student`s t test p<0.05 n=10.

**Control**		
Col-0	43 ± 5 a	147 ± 16 a
ttl1	33± 5 b	101 ± 15 b
prc1-1	27± 5 c	48 ± 8 b
ttl1prc1-1	27 ± 2 c	38 ± 9 c

Table S 6. Cortical cell number in the PM and Mature cortical cell length meassured at -0.7 MPa. The tables show the number of cortical cells in the proximal meristem and the cortical mature cell length for Col-0, *ttl1, prc1-1 and ttl1prc1-1* grown during 7 days in -0.7MPa osmotic stress conditions. Values are mean and SD. Different letters indicates statistically significant differences; Student`s t test p<0.05 n=10.

**-0.7 MPa**		
Col-0	30 ± 4.4 a	79.8 ± 13.6 a
ttl1	25± 3.9 b	47.9 ± 8.8 b
prc1-1	29 ± 3.7 a	50.8 ± 8.1 b
ttl1prc1-1	21 ± 3.1 c	39.3 ±8.2 c

Table S 7. Cortical cell number in the PM and Mature cortical cell length meassured at -1.2 MPa. The tables show the number of cortical cells in the proximal meristem and the cortical mature cell length for Col-0, *ttl1, prc1-1 and ttl1prc1-1* grown during 7 days in -1.2 MPa osmotic stress conditions. Values are mean and SD. Different letters indicates statistically significant differences; Student`s t test p<0.05 n=10.

**-1.2 MPa**		
Col-0	12 ± 1.4 a	33.5 ± 10.6 a
ttl1	9 ± 2.5 a	39.3± 6.3 a
prc1-1	10 ± 1.3 a	36.1 ± 9.5 a
ttl1prc1-1	5± 3.1 b	27.6 ± 8.8 a

Table S 8. Root length of Col-0 and *ttl1* after 9 days of growth in medium with, 0.2 μM of BL, 0.5 μM of PCZ, without or with supplementation of 400 mM.

Root length (cm)<br>Root length reduction effect →								
	Control				400mM Mannitol			
		0.5 μM PCZ	0.1μM BL	0.2μM BL	0.2 μM BL	0.1μM BL	0.5 μM PCZ	
Col-0	2,83 ± 0,66	2,25 ± 0,37	2,3 ± 0,25	2,04 ± 0,17*	2,21 ± 0,21*	1,83 ± 0,18*	1,73 ± 0,38*	1,56 ± 0,32*
ttl1	2,79 ± 0,13	1,91 ± 0,23*	1,87 ± 0,19*	1,63 ± 0,21*	1,6 ± 0,16*	1,26 ± 0,19*	1,16 ± 0,26*	1,34 ± 0,34*

* Indicates statistically significant differences with Col-0 grown in control medium; Student`s t test p<0.05, n=7 and cells with different colors indicates treatments with significantly different effect, the intensity of gray indicates the degree of root length reduction.

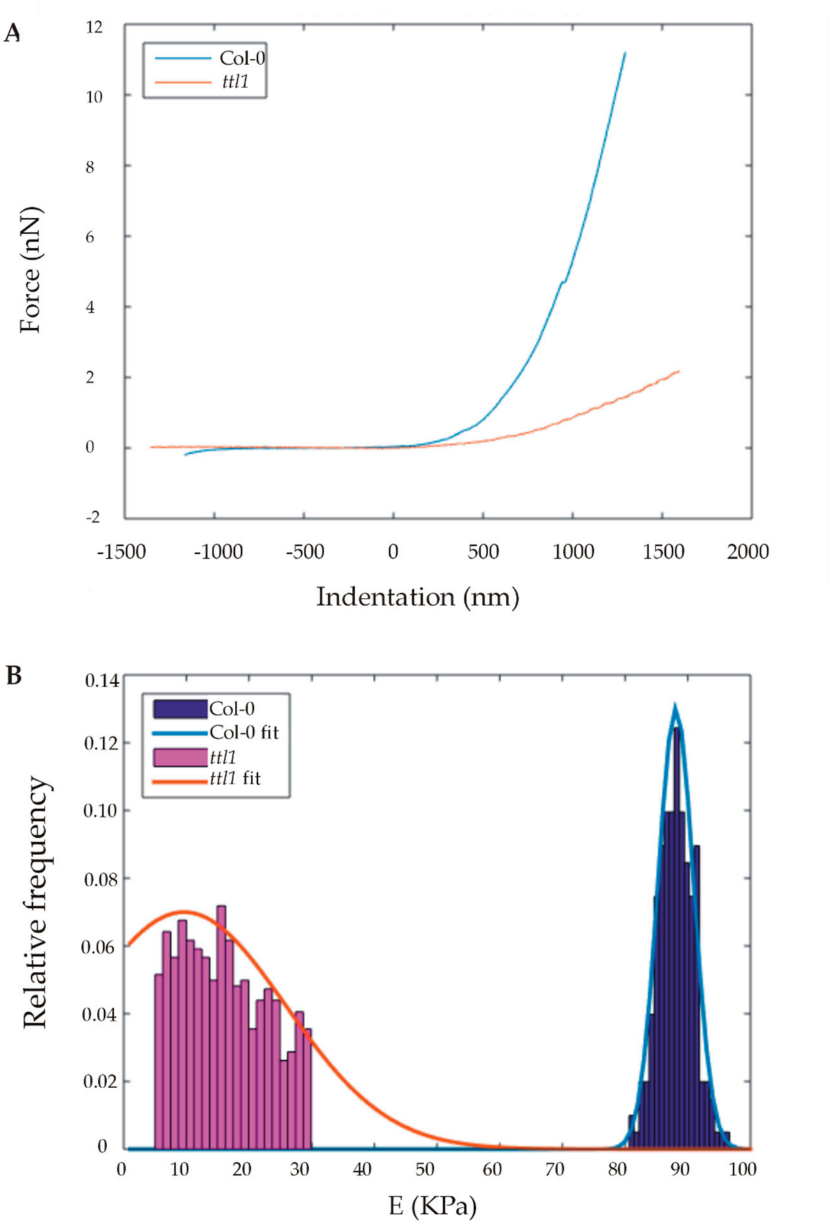

Figure S 1. Force curves and apparent elastic modulus (AEM) of cell walls of epidermal cells in the EZ of Col-0 and *ttl1* roots grown in control conditions.**A:** Representative force (F) vs indentation curves on Col-0 (blue line) and *ttl1* (red line) roots grown in control conditions. From each force curve, a value for AEM is calculated. **B:** histograms for AEM of Col-0 and *ttl1* grown in control conditions, with the corresponding Gaussian fit for each condition. The mean and standard deviation values of each condition presented in Figure 2 C was obtained from the Gaussian fits. The data were collected from 3 roots (201 curves for Col-0 and 1184 curves for *ttl1*).

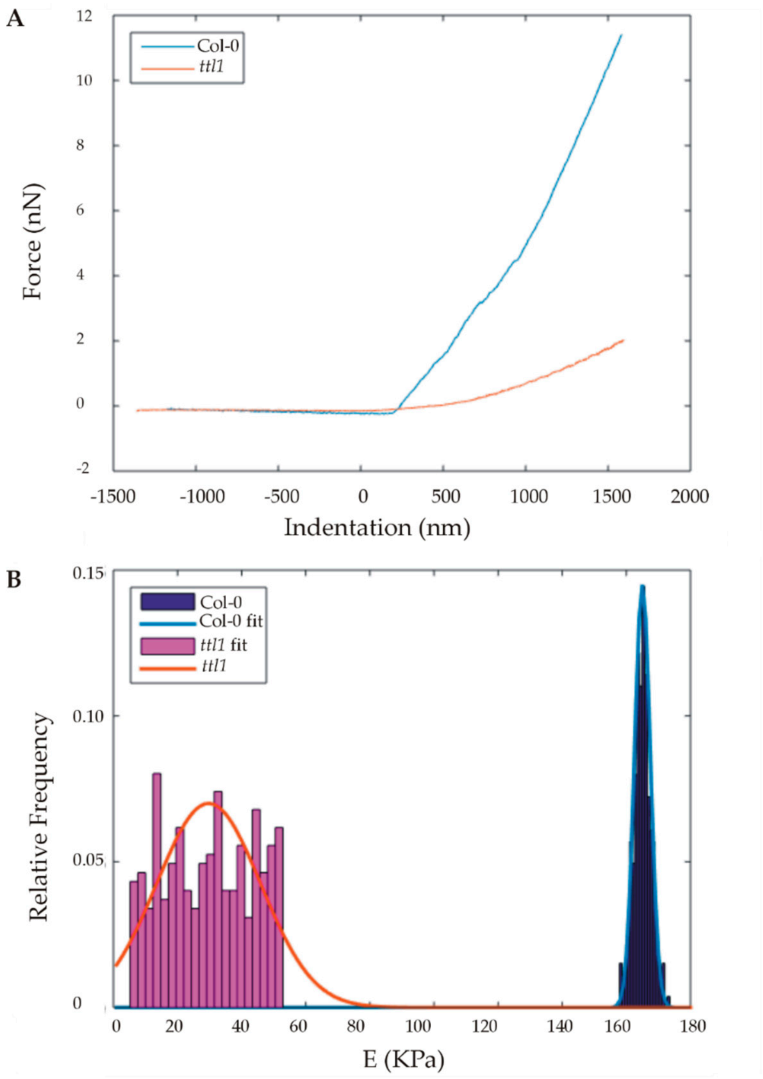

Figure S 2. Force curves and apparent elastic modulus (AEM) of cell walls of epidermal cells in the EZ of Col-0 and *ttl1* roots grown under osmotic stress. **A:** Representative force (F) vs. indentation curves of Col-0 (blue line) and *ttl1* (red line) roots grown under osmotic stress conditions. From each curve, a value for AEM is calculated. **B:** histograms for AEM of Col-0 and *ttl1* grown under osmotic stress conditions, with the corresponding Gaussian fit for each condition. The mean and standard deviation values of each condition presented in Figure 2 C was obtained from the Gaussian fits. The data were collected from 3 roots (263 curves for Col-0 and 324 curves for *ttl1*).

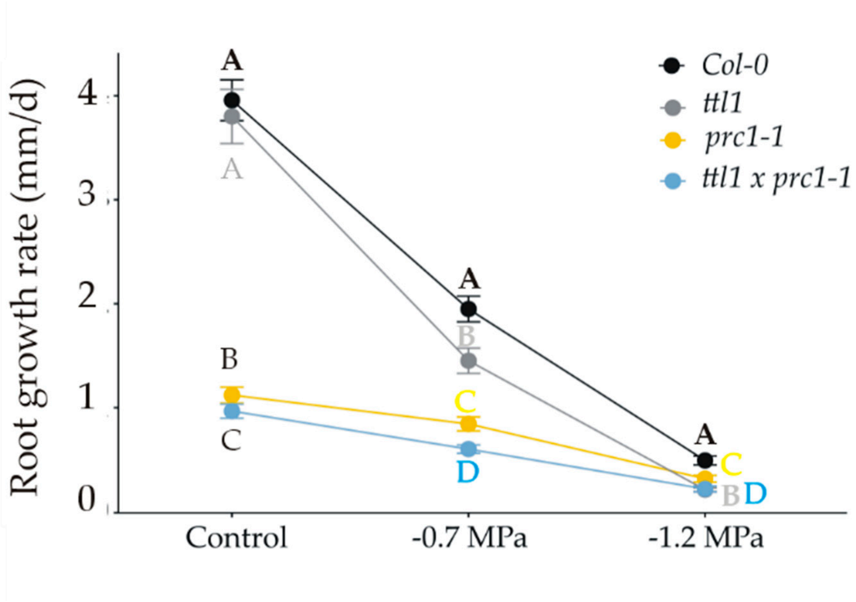

Figure S 3. The *ttl1prc1-1* mutant exhibits greater reduction of root growth rate than Col-0 in response to increasing osmotic potential**. A.** The graphic shows the root growth rate for Col-0, *ttl1, prc1-1 and ttl1prc1-1* grown during 10 and 7 days in control and stressing -0,7 MPa and -1,2Mpa conditions, respectively. The magnitude of deceleration is higher in *ttl1prc1-1.* Different letters indicate statistically significant differences (t Student p < 0.001; n = 30).

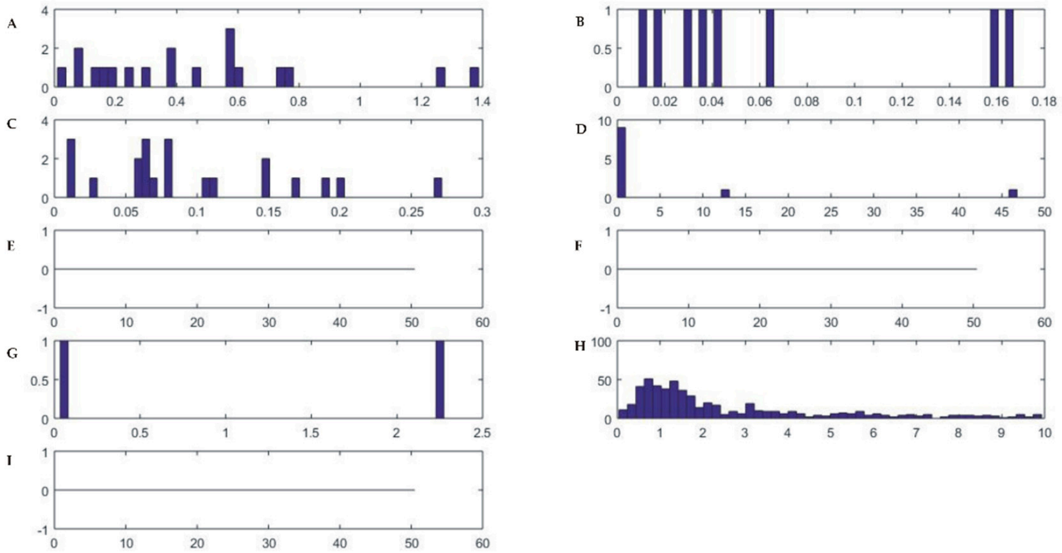

Figure S 4. Histogram of Young`s modulus obtained from force curves on 9 different cells of three different plants of the *ttl1prc1-1* double mutant. On 8 of the 9 cells, force curves obtained did not allow proper analysis; only histogram H allowed proper analysis.

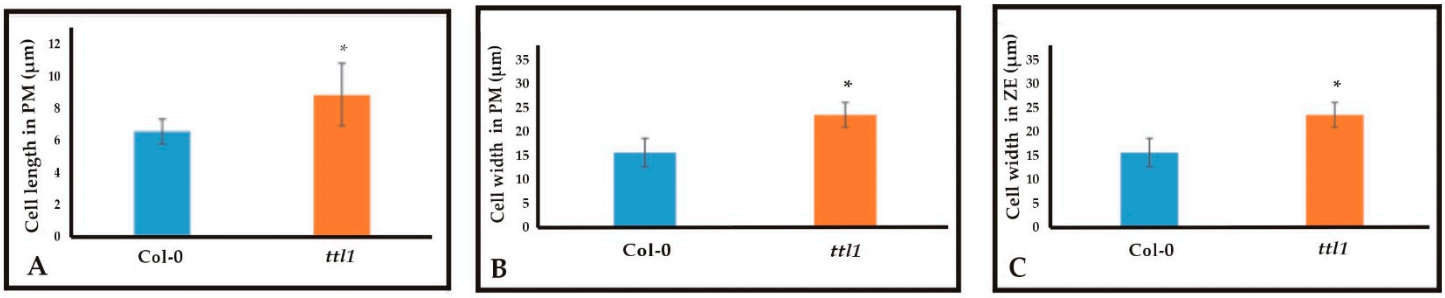

Figure S 5. Cell size in the PM and EZ determined in control conditions for Col-0 and *ttl1* mutant. A. Cell length in PM; B. Cell width in PM and C. Cell width in ZE. * Indicates values that are statistically different; Tukey, $p<0.05$. n= 20.

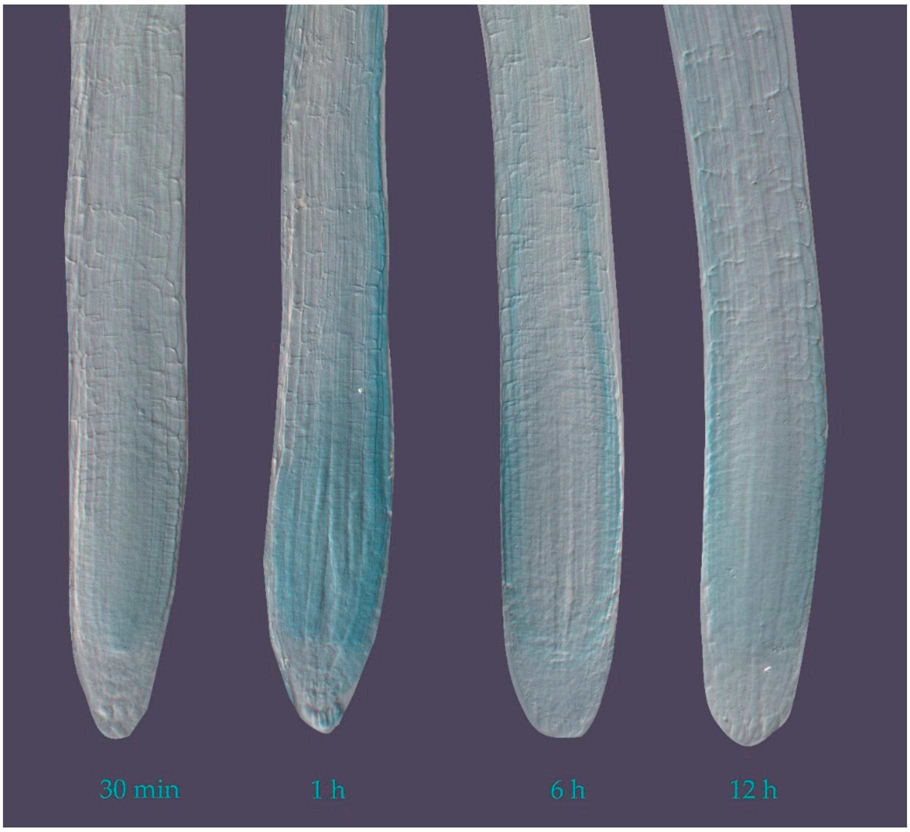

Figure S 6. *pTTL1::GUS* signal in response to different time of osmotic stress (-1.2 MPa).
